# Systematic review to understand users perspectives on AI-enabled decision aids to inform shared decision making

**DOI:** 10.1038/s41746-024-01326-y

**Published:** 2024-11-21

**Authors:** Nehal Hassan, Robert Slight, Kweku Bimpong, David W. Bates, Daniel Weiand, Akke Vellinga, Graham Morgan, Sarah P. Slight

**Affiliations:** 1 School of Pharmacy / Population Health Sciences Institute, Newcastle University, Newcastle upon Tyne, United Kingdom; 2 Newcastle Upon Tyne Hospitals NHS Foundation Trust, Freeman Hospital, Newcastle upon Tyne, UK; 3 Department of General Internal Medicine, Brigham & Women’s Hospital, Harvard Medical School, Boston, MA USA; 4 School of Medicine, University College Dublin, Belfield, Dublin Ireland; 5 School of Computing, Newcastle University, Urban Sciences Building, Newcastle upon Tyne, UK

**Keywords:** Epidemiology, Health care, Diseases

## Abstract

Artificial intelligence (AI)-enabled decision aids can contribute to the shared decision-making process between patients and clinicians through personalised recommendations. This systematic review aims to understand users’ perceptions on using AI-enabled decision aids to inform shared decision-making. Four databases were searched. The population, intervention, comparison, outcomes and study design tool was used to formulate eligibility criteria. Titles, abstracts and full texts were independently screened and PRISMA guidelines followed. A narrative synthesis was conducted. Twenty-six articles were included, with AI-enabled decision aids used for screening and prevention, prognosis, and treatment. Patients found the AI-enabled decision aids easy to understand and user-friendly, fostering a sense of ownership and promoting better adherence to recommended treatment. Clinicians expressed concerns about how up-to-date the information was and the potential for over- or under-treatment. Despite users’ positive perceptions, they also acknowledged certain challenges relating to the usage and risk of bias that would need to be addressed.

**Registration:** PROSPERO database: (CRD42020220320)

## Introduction

**Fig. 1:**
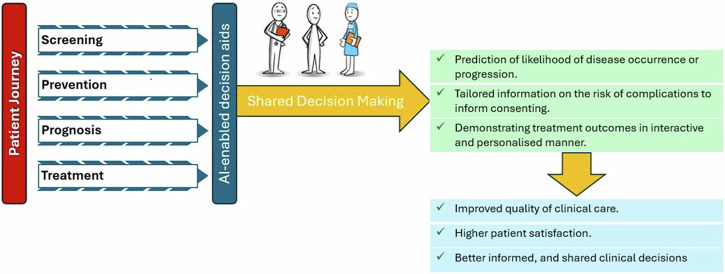
The inter-relationship between AI-enabled decision aids, shared decision-making, and the patient journey. The figure describes how AI-enabled decision aids could inform shared decision-making across different stages of patient journey.

Shared decision-making (SDM) represents a collaborative process through which a clinician supports a patient to make a decision that is right for them^1^. These decisions can occur at different stages of the patient journey, such as when deciding on a suitable treatment option^2^. There are different types of decision aids that are frequently used to assist SDM^3^. Some use in-built artificial intelligence (AI) functionality to predict the likelihood of a disease occurring or progressing such as the direct-to-patient AI tool for skin cancer screening^4^, or provide tailored information on the risk of complication(s) after undergoing a particular medical procedure such as the American College of Surgeons Risk Calculator^5^. Compared to conventional decision-aids, an AI-enabled decision aid is developed through learning from patterns in real patients data and health outcomes that resulted from a particular intervention or treatment. This learning process allows AI-enabled decision aids to predict risks and/or tailor treatment plans to individual patients, which can be presented to both patients and/or their care providers in the form of interactive risk scalers^6^, graphs or charts^7,8^. This can help enrich the conversations between patients and their clinicians^9^. Fig. 1 provides a visual representation of how AI-enabled decision aids facilitate shared decision-making during the patient journey.

Unlike traditional information technology, AI-enabled tools have the ability to provide more personalised information to patients to help inform the SDM process. AI systems can learn patterns from large healthcare data and use sophisticated algorithms to predict the likely health outcome or suggest a more suitable course of action for the patient, which is often lacking with traditional computing^10^. Accordingly, it is important to understand patients’ and clinicians’ perspectives on the use of these AI-enabled decision aids^11^. These include the benefits of these tools, such as allowing more time for doctor-patient discussion, and reducing anxiety among surgical patients by providing detailed personalised information about the expected procedure and post-surgical recovery time^12^. However, AI-enabled decision aids also have some limitations such as, for example, that patients and/or clinicians may be unable to understand the presented information, or the decision aid itself cannot be integrated with larger electronic healthcare systems^13^. We conducted a systematic review of the literature to understand clinicians’ and patients’ perspectives on using AI-enabled decision aids to support shared decision-making at different stages of the patient journey and in various clinical settings.

## Results

A total of 509 research articles were identified in October 2020, and duplicates (*n* = 12) removed. Articles were screened at the title (*n* = 497), abstract (*n* = 374), and full-text (*n* = 19) stages, with 17 articles eligible for inclusion^7,12,14–28^. This search was updated in February 2022 and an additional nine articles included, giving a final total of 26 articles^6,8,29–35^. These included studies used either quantitative, qualitative or mixed methods (i.e., model development and perceptions exploration). Nine qualitative studies used either face-to-face interviews^6,15,16,24,30^, focus groups^22,25^, or a combination of interviews and telephone follow-up calls^16,31^. Fifteen studies used surveys,^7,8,12,18,20,21,23,26–29,31,33–35^, nine of which collected text answers^18,20,23,26,28,29,31,33,35^. Two mixed-method studies used a combination of survey and semi-structured interviews^14,28^.

In terms of the AI-enabled decision aids, 12 studies developed and/or evaluated web-based tools^6–8,12,17,19,21,22,26,29–31^, three studies utilised smart phone and tablet applications^32–34^, and 11 studies evaluated aids that were developed and embedded in larger electronic systems such as Cerner^14–16,18,20,23–25,27,28,35^. Table 1 illustrates the name, type, and purpose of each AI-enabled decision aid, and contribution to SDM. Ten studies explored patients’ perceptions only^6,8,12,15,17,29,31–34^, eight studies clinicians’ perceptions only^7,14,18,21,22,24,25,35^, and the remaining eight studies both clinicians’ and patients’ perceptions.^16,19,20,23,26–28,30^.

**Table 1:** Description of AI-enabled decision aids

Study	Name	Type	Medical speciality	Clinical setting	Purpose	Contribution to SDM
Ballard et al.^7^	Statin Choice Decision Aid (SCDA) and the Diabetes Medication Choice Decision Aid (DMCDA)	web-based	Internal medicine (Diabetes)	Mayo Clinic, an academic tertiary healthcare centre	To estimate 10-year cardiovascular risk, the degree of risk reduction with a statin, and the likelihood of adverse events (SCDA) To estimate the impact of different diabetes medications on daily routine, blood sugar control, risk of hypoglycemia, weight change, and cost (DMCDA)	Clinicians accessed the tools through EMR during the consultations and share the recommendations from the tool with the patient in the form of graphs which inform the medication choice based on their risks and benefits.
Raymond et al.^12^	Surgical Risk Calculator (ACS Calculator)	web-based	Surgery	Pre-operative clinic	To provide patient-specific risks for common adverse postoperative outcomes	Informed the consenting process between the patient and clinician with more detailed information on risks.
Manski-Nankervis et al.^14^	No name specified	Embedded in electronic health system	Infectious diseases	Primary care	To guide the treatment of infections (e.g., antibiotic choice, dose, duration)	Educated patients about potentially inappropriate antibiotic prescribing, when clinicians shared them with patients during consultation.
Nelson et al.^15^	Direct-to-patient AI tool (DTP AI tool)	web-based	Dermatology	General dermatology clinic	To provide the patient with information on skin cancer screening options	Increased patients’ understanding and helped them to better frame their questions to clinicians.
Qu et al.^16^	SMILE	web-based	Dermatology	Lupus clinic	To educate patients about Lupus treatment during a regular clinic visit	Helped patients learn more about the disease and inform their treatment options during clinical consultations.
Eckman et al.^17^	Atrial Fibrillation Shared Decision Making Tool (AFSDM)	web-based	Cardiology	Arrhythmia clinic	To generate patient-level recommendations for thromboprophylaxis	Clinicians use the tool with patients during consultation and show them the recommendations which improved patients’ understanding about risks and benefits between different anticoagulation options.
Brown et al.^18^	Clinical prediction models (CPM)	Non-cardiovascular CPMs (different types: Web-based/Software/embedded into systems)	Family medicine	North West England, NHS North Western Deanery	Prognosis of non-cardiovascular long-term conditions	Increased patients understanding on prognosis with different treatment options to aid further discussions with the clinician.
Silvestrin et al.^19^	Women’s Health Assessment Tool/ Clinical Decision Support toolkit (WHAT/CDS toolkit)	web-based	Women’s health	Two primary care and one gynaecology clinics	To assess depression, vulvovaginal atrophy and provide tailored evidence-based treatment recommendations	Clinicians used the tool during consultation with patients to show patients the benefits and risks of their treatment options based on their own symptoms and help them decide on their treatment.
Flynn et al.^20^	Computerised decision Aid for Stroke thrombolysis (COMPASS)	iPad based software	Neurology (Stroke)	Stroke units	To predict the patient-specific probability of acute stroke outcomes at three months, with and without thrombolysis	Used by the clinician with the patient or relative upon discharge from stroke unit to decide on thromboprophylaxis management.
Schroy III et al.^21^	CRC screening decision aids	web-based	Oncology (Colorectal cancer)	Primary care (screening) clinic	To convey key information about colorectal cancer, and the importance of screening	Patients used the tool before their consultation to understand more about their tailored screening options, which they take to discuss with the clinician.
Jimbo et al.^22^	CRC screening decision aids	web-based	Oncology (Colorectal cancer)	Community practice	To inform decision making around the preferred colorectal cancer screening method	Educated patients about the disease and different screening programmes available before the consultation, so that they have more focused questions to their clinicians about the screening methods they preferred.
Schackmann et al.^23^	Decision tool for women with BRCA mutation	Embedded into electronic system	Oncology (Breast cancer)	Community practice	To predict the risk of breast cancer and mortality in BRCA1/2 mutation carriers	Tailored the cancer prevention strategies for the patients based on their own needs before the consultation to discuss their best strategy with their clinician.
Cunich et al.^24^	ALProst embedded in Annalisa (AL)	web-based	Oncology (Prostate cancer)	Primary care	To predict prognosis and diagnosis of prostate cancer from prostate antigen test and patient’s age.	Used during consultation to summarise risk profile of patients to the GP and provide patients with visuals to understand their prognosis.
Harrison et al.^25^	Annalisa, Computer decision aid and paper-based decision aid (3 decision aids)	web-based	Surgery	University of Sydney and Sydney South West Area Health Service	To calculate the risk estimates of surgical treatment of rectal cancer in terms of complications and outcomes	Used in pre-operative assessment setting to guide the conversation about surgery consent between surgeon and patient through tailored assessment of surgical risk.
Aoki et al.^26^	u-SHARE	web-based	Neurosurgery	Neurosurgery clinics	To provide favourable treatment options, and critical factors for decision making on unruptured intracranial Aneurysms.	Used during consultation to help clinicians explain to patients the difference between pharmacological and non-pharmacological options tailored to each patient, so that they can decide which option to go with.
Siminoff et al.^27^	Adjuvant!	embedded into electronic system	Oncology (Breast cancer)	Community Practices	To assist in adjunctive therapy choices for breast cancer.	Clinicians used the tool over the EMR during patients consultation to guide their conversation with patients about different breast cancer adjunctive treatments suitable for them to choose from.
Thomson et al.^28^	DARTs tool	embedded into electronic system	Neurology (stroke prevention)	General practitioner practices	To support shared decision making in atrial fibrillation, deciding on whether to take warfarin to prevent stroke.	Used during clinics to guide prophylaxis anticoagulation discussion between patients and clinicians.
Berry et al.^29^	Personal Patient Profile- Prostate (P3P)	web-based	Urologic oncology (prostate cancer)	Urology and radiation outpatient clinics	To guide patient-provider communication tailored to race and age.	Patient used the tool before the consultation to enter their preferences and values towards prostate cancer and clinicians receive a printout of the results to use with the patient during consultation.
Jones et al.^6^	RealRisks	web-based	Oncology (Breast cancer)	Community Practices	To promote a woman’s understanding of her breast cancer risk and to engage women in planning a tailored chemoprevention plan.	Patients visit the website before their clinics to tailor the chemoprevention plan and understand its 6-months outcome, they take the results for discussion with clinician during their consultation.
Kim et al.^30^	myAID	Web-based, Australianised version of original US decision aid (UCTO- Ulcerative Colitis Treatment Options)	Gastroenterology (Ulcerative colitis)	Outpatient clinic	To facilitate and support SDM for treatment decisions in ulcerative colitis pharmacological management	Provided patients with tailored information about treatments suitable for them before consultation to discuss with clinicians in the clinics.
Kosch et al.^8^	Evidence-Based Decision Support Tool in Multiple Sclerosis’ (EBDiMS)	web-based	Neurology (Multiple Sclerosis)	Multiple Sclerosis Day clinic	To provide an estimation of the individualised long-term prognosis (up to 30 years)	Provided patients with better understanding about what to expect with their disease in the future, with information that was relevant to their own symptoms through plots and graphs that patients take to their clinicians for discussion in follow-up clinics.
Lau et al.^31^	shouldiscreen.com	web-based	Oncology (Lung Cancer)	Community-based organisations	To provide basic information about low-dose computed tomography screening, education about lung cancer risk factors, and a lung cancer risk calculator that computes a personalised risk	The patients use the website to get a personalised risk of their lung cancer based on their demographics, social and medical history. They take the results from the website to their screening appointment to discuss the results with their clinician.
Kuppermann et al.^32^	PROCEED	tablet-based	Gynaecology (delivery mode and approach)	prenatal clinics	To guide decision making on trial of labor after caesarean (TOLAC) versus caesarean section.	Used in prenatal clinic to inform consenting process between patients and surgeons before the procedure.
Weismann et al.^33^	No name specified	hypothetical models to be embedded into electronic records	Respiratory (Chronic Lung Disease)	outpatient pulmonary clinic	To estimate future risk of in-hospital death with advanced chronic lung disease.	This hypothetical tool was proposed to be used during clinics and accessed by clinicians through the EMR to discuss disease control with patients.
Wilson et al.^34^	No name specified	Smart phone application	Internal medicine (Diabetes)	academic medical centre’s diabetes clinic	To guide decisions on treatment for type 1 diabetes	Guided clinicians-patients conversations during consultation on the tailored management of hypoglycaemia.
Coylewright et al.^35^	HealthDecision	Embedded in electronic health system	Multiple	Outpatient clinic	To calculate and display personalised risk estimates for cardiovascular risk, stroke prevention for atrial fibrillation, fracture prevention in osteoporosis, and breast and lung cancer screening)	Used during consultation where clinicians share with patients charts from the tool showing their risk reduction with treatment, to guide conversation about disease prophylaxis.

**Fig. 2:**
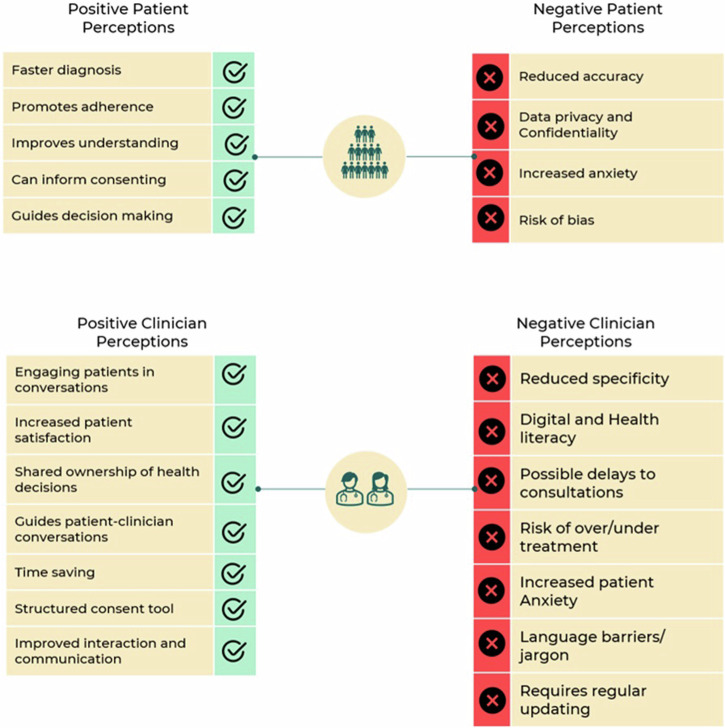
Key themes emerging from both patients’ and clinicians’ perceptions. Summary of key themes describing the perceptions of patients (upper section) and clinicians (lower section) towards AI-enabled decision aids.

The majority of studies (*n* = 22) were conducted in a medical setting^6–8,14–24,27–31,33–35^, and four were done in a surgical setting^12,25,26,32^. All AI-enabled decision aids were used to guide SDM process at different stages of the patient journey, including screening and prevention (risk prediction)^6,15,17,18,21,22,24,31,35^, prognosis^7,8,12,23,24,26,33^, and treatment^7,14,16,19,20,25,27–30,32,34^, as illustrated in Table 2. We discuss each of these different stages in further detail below. We also categorised the key themes to offer deeper insights into the specific users’ experiences in Fig. 2.

**Table 2:**
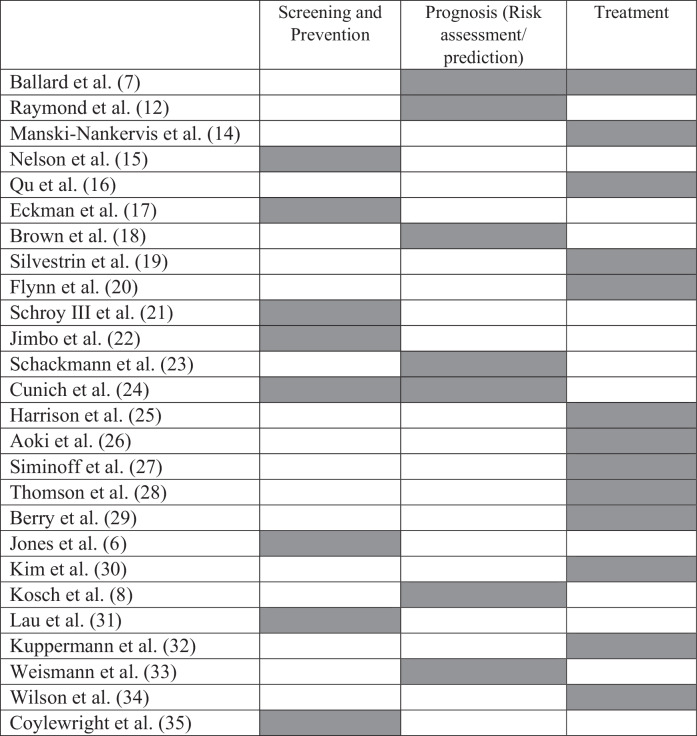
Role of artificial intelligence decision aids at different stages of the patient journey

### Screening and prevention (risk prediction)

Ten studies evaluated the perceptions of either patients and/or clinicians on AI-enabled decision aids for screening (*n* = 6)^15,21,22,24,31,35^, and prevention (*n* = 4)^6,17,18,35^.

#### Screening

The *Direct-To-Patient (DTP)* AI tool scanned images of skin lesions submitted by patients before attending their general dermatology clinics in Boston, Massachusetts, United States, and estimated their likelihood of having skin cancer^15^. Sixty percent (*n* = 29) of patients felt that *DTP* increased diagnostic speed, with one patient explaining how *“If the app says, ‘you probably have melanoma—go see your doctor,’ they might actually get in there sooner…so it could be lifesaving”*^15^. However, 85% (*n* = 41) expressed their concerns about the accuracy of the tool when coupled with the lack of a physical examination. One patient highlighted how: *“[AI] is not a substitute for [an] in-person exam. We’re examining a photograph that you get with your phone in variable light. It’s possible that you might get a false-positive or a false-negative.”*^15^ A second AI-enabled decision aid, *ALProst®*, offered personalised insights on the benefits and risks of prostate cancer screening for an individual patient, based on their prostate-specific antigen (PSA) test result and age^24^. Clinicians described how this information was displayed in an understandable format, which helped patients engage with the tool and gave them a sense of ownership^24^. However, 80% (*n* = 8) of clinicians reported challenges with its use, including how the tool did not incorporate patient-specific cardiovascular risk factors^24^. A second AI-enabled decision aid in the area of cancer screening compared various colorectal cancer screening options for patients based on their cost, discomfort, inaccuracy, and requirement for additional tests. This decision aid also obtained the patient’s preferred screening option and shared this with their clinician prior to their consultation^22^. Clinicians perceived an increase in patients’ satisfaction amongst those who completed the tool; however, others expressed their concerns about patients using the tool in the waiting area, in particular patients’ digital literacy, the potential for theft and damage to the device that the patient is using, and a knock-on delay to them attending their appointments^22^.

“*[many patients have] no comfort with computers, and I can’t see more than perhaps 15, 20% of our total patient population even being able to access that. And of that, [we would be lucky] if we had 5 or 7% that would actually do it to the level that you are discussing….[F]or a certain group of patients that would be a wonderful tool. I don’t think it would be for our group of patients primarily because we have a large percentage of low functioning patients*”^22^.

#### Prevention

The Atrial Fibrillation Shared Decision-Making aid recommended the most suitable type of thromboprophylaxis (i.e. warfarin versus DOACs) for individual patients. It also presented information on intracranial and extracranial bleeding risks, which patients found helpful and promoted their adherence to recommended treatments (*p* < 0.0001) after one month^17^. The web-based AI-enabled decision aid, *RealRisks®*, provided women with a personalised risk score of developing breast cancer over the next 5-years, along with chemo-preventive measures to print out and discuss with their healthcare provider during their consultation^6^. Although all participants (*n* = 21) deemed this tool *“helpful”* and *“acceptable”*, 43% (*n* = 9) of patients requested clearer, jargon-free information so as to improve their understanding, and felt that *RealRisks®* put a heavy emphasis on chemoprevention^6^. The aid was also perceived as tailored to Hispanic/Latina individuals, with (43%, *n* = 9) of participants expressing their concerns about the tool’s suitability for other ethnicities^6^.

Coylewright et al.^35^ examined clinicians’ perceptions of an integrated patient decision aid (*iPDA)*, which predicted the risk of a patient developing various conditions (atrial fibrillation, cardiovascular disease, fractures with osteoporosis, and lung and breast cancer) based on their demographics, medical history, and preference for preventive measures^35^. Over 80% (*n* = 266) of clinicians used *iPDA* in the last three months to guide their patient-clinician conversations and found it time-saving^35^. However, family physicians shared their concerns about another AI-enabled decision aid, which predicted the risk of a patient developing non-cardiac conditions, explaining how the tool could influence over- or under-treatment of patients, mainly due to specific risk factors not being considered^18^. These physicians also felt the tool could increase anxiety amongst patients who had a high mortality or morbidity risk, and so in-turn might have chosen not to receive the information^18^.

### Prognosis

Five studies incorporated AI-enabled decision aids to predict disease prognosis and/or the likelihood of a particular complication(s)^8,12,23,26,33^. The Evidence-Based Decision Support Tool in Multiple Sclerosis (*EBDiMS*) provided information on the long-term prognosis of multiple sclerosis (over 30 years), which participants perceived as *“relevant”* and *“highly understandable”*^8^. Ninety-five percent (*n* = 85) of participants would recommend this decision aid to others, with 54% (*n* = 48) highlighting how they would have liked earlier access to the tool itself^8^. A second web-based Surgical Risk Calculator (*ACS Calculator*) predicted post-operative complications, with 90% (*n* = 135) of patients perceiving it as particularly useful during the surgical consenting process^12^. Seventy percent (*n* = 105) of patients were willing to participate in a pre-habilitation programme to lower peri-operative risk after obtaining their risk score, with 38.6% (*n* = 58) considering postponing their surgery until after they had participated in this programme^12^.

The u-SHARE decision aid also provided patients with a personalised risk of post-operative complications following surgery, when compared to watchful waiting^26^. Over half of the patients (*n* = 8) were comfortable using the tool. However, acceptance appeared to be higher amongst patients than clinicians, with clinicians perceiving the decision aid as a *“structured informed consent tool”*^26^. In contrast, oncologists reported higher satisfaction and acceptance with another AI-enabled decision aid that provided information about different breast cancer mutations and preventative measures than patients. Oncologists perceived the use of this decision aid improved their interaction and communication with patients during the consultation, with patients finding illustrative graphics and visual aids particularly beneficial^23^.

#### Treatment

Twelve studies explored patients’ and clinicians’ perceptions of AI-enabled decision aids used for pharmacological or non-pharmacological decisions (i.e. surgical procedure)^7,14,16,19,20,25,27–30,32,34^. The COMPASS decision aid and Decision Analysis in Routine Treatment Study (DARTs) tool outlined the benefits and risks of thrombolytic therapy^20,28^. While patients found these personalised risk estimates helpful, some had negative feelings around receiving such information^20^. Clinicians noted that patients’ health literacy levels, personalities, and preferences influenced their engagement with COMPASS and the SDM process^20^. Some clinicians also expressed reservations about whether DARTs’s recommendations incorporated the latest guidance^28^. Similar concerns were raised about the information provided by the Diabetes Medication Choice Decision Aid (DMCDA), which clinicians perceived as somewhat non-relevant and not evidence-based^7^. Only 28% (*n* = 30) of clinicians routinely used the DMCDA tool compared to 80.6% (*n* = 75) who used the Statin Choice Decision Aid (SCDA), a decision aid that also supplied personalised benefits and risks of statin therapy^7^.

The AI-enabled decision aid *Annalisa®* presented patients with personalised risk estimates of developing side-effects from drug treatment (medical option) for rectal cancer versus complications from post-surgery (surgical option)^25^. Patients felt that *Annalisa®* presented the information clearly and improved their understanding of their clinical condition, in contrast to 25% (n = 5) of colorectal surgeons who perceived *Annalisa*® as lacking specific information. Surgeons also raised concerns about the time needed for patients to use the tool and felt it was *“designed for those who speak English”*^25^.

Another decision aid, which displayed estimates of a patient’s 10-year mortality risk with a surgical intervention versus non-surgical intervention for breast cancer *(Adjuvant*®*)*, was perceived by 48% (*n* = 169) of patients as guiding their decision-making on adjuvant therapy and by 75% (*n* = 43) of clinicians as helping them develop more individualised treatment protocols for their patients^27^. Similarly, the Women’s Health Assessment Tool/Clinical Decision Support toolkit (*WHAT)*, which offered evidence-based treatments for menopausal symptoms, was perceived by 68% (*n* = 75) of patients as improving the quality of healthcare provided and by 25% (*n* = 3/12) of clinicians as helping them to “*get right to the point and the patient feels valued. And they feel a part of their healthcare (…) plan*”^19^. More than half of clinicians (*n* = 5/8) were “*neutral*” or “*somewhat agreed*” that the WHAT toolkit delivered more efficient clinical care^19^. GPs (*n* = 7) found another simulated AI-enabled decision aid valuable as they could print out the specific guideline on antibiotic treatment and share it with the patient during their consultation. One GP felt that the tool was less valuable and only used it when they were uncertain about the treatment: *“you’re basically looking at the computer while you’re talking to a person in there, and so you look a bit like an idiot, really”*^14^.

Another AI-enabled decision aid, which provided clinicians with comprehensive information on the different formulations, costs, doses and side effects of immunosuppressive agents for the treatment of lupus, was found to be useful during consultations as it helped encourage patients to discuss any concerns about their treatment and feel more confident about their treatment plan, potentially improving adherence^16^. However, 48% (*n* = 12) of participants (including physicians, clinic champions and patient advocates) raised concerns about privacy and confidentiality when asked to complete this tool in the patient waiting room^16^.

Finally, AI-enabled decision aid in the form of smartphone application alerted type 1 diabetic patients either when their insulin doses were due and/or 30 min before a hypoglycaemia event was likely to occur. Over 78% (*n* = 1202) and 64% (*n* = 986) of patients used the tool to guide rapid-acting and long-acting insulin doses, respectively^34^. Although 91% (n = 1403) of patients were particularly interested in the ability of the tool to help them avoid hypoglycaemia, 85% (*n* = 1310) asked if the hypoglycaemia alert feature could be further customised based on their blood glucose levels over time^34^.

## Discussion

We explored how patients and clinicians perceived the use of AI-enabled decision aids in supporting SDM. Twenty-six articles were included^6–8,12,14–35^, the majority of which focused on either patients and/or clinicians’ perceptions of using decision aids in the medical setting (*n* = 22). AI-enabled decision aids were either web-based tools, smart phone applications or integrated within larger electronic systems, and used at different stages of the patient journey, including screening and prevention (risk prediction), prognosis, and treatment. Patients generally found the AI-enabled decision aids easy to understand and user-friendly. This helped increase their engagement with the tool, fostering a sense of ownership and promoting adherence to recommended treatments^8,17,24,25^. Some clinicians perceived AI-enabled decision aids facilitated the patient consent process for procedures or treatments^12,26^, and felt patients’ interaction with tools was influenced by their health literacy, personalities, and preferences^20,35^. There was a recognised risk of bias associated with AI-enabled decision aids with variations in patients’ usage, possibly due to their different levels of health and technological literacy^6,16,17,20,30^. This bias could potentially put some patients at risk of digital exclusion and exacerbate digital health inequity^36^. Bias could also lead to underdiagnosis among some ethnicities, possibly delaying access to clinical care^37^. For example, a widely used algorithm falsely concluded that patients from Black ethnicities were healthier than equally sick White patients, thus reducing the number of Black patients identified for extra care^37^. Clinicians expressed concerns about how up-to-date the decision aid information was^28^, and the potential for over- or under-treatment^8,15,21^. The included studies used AI-enabled decision aids mostly for screening purposes, with more perceived challenges reported with their application in treatment and prevention.

A recent scoping review supported these findings reporting patients’ positive perceptions on the ease of use of AI tools in general and their potential to enhance efficiency^38^. Another review on the application of AI-enabled decision aids specifically to support SDM described how these aids are mainly applied in primary and secondary care settings, rather than tertiary care settings. However, this review included only six studies, and did not explore users’ perceptions^39^. In the United States, a recent systematic review examined the attitudes of the public on using AI more broadly in healthcare and highlighted similar concerns around data confidentiality and the use and security of their healthcare data, as highlighted in this review^40^. Another systematic review also identified organisational, patients and professional characteristics that influenced the adoption of AI generally in healthcare, demonstrating that; psychosocial factors such as ease- of- use and usefulness were the common determinants of adopting AI in healthcare adoption^41^. Additionally, Young et al. systematically reviewed the attitudes of patients and general public towards AI in clinical care^42^. However, SDM is a collaborative process between patients and their clinicians^1^; our systematic review included both patients’ and clinicians’ perceptions, which is important, and also the key themes (both positive and negative) that emerged (see Fig. 2), which offers a deeper insights into the specific users’ experiences.

As AI-enabled decision aids become integrated into various aspects of healthcare, it is crucial that they do not perpetuate any existing health inequities for minority ethnic groups. Johnes et al highlighted how an AI-enabled decision aid tested among Hispanic/Latina individuals might lead to racial bias by excluding other ethnic groups^6^. Jimbo et al also showed that with a web-based tool, concerns were raised by clinicians about the affordability of these devices alongside patients’ ability to access the internet and their familiarity with technology. These barriers can contribute to unequal access to healthcare, worse experiences of healthcare, and wider inequities in society^22^.

The suitability of the information displayed by decision aids was also raised^6,17^, with Jones et al. and Eckman et al. suggesting that the display be customised to accommodate the needs of elderly patients and those with visual impairments. Patients found the illustrative graphics and visual aids for a prognostic decision aid particularly beneficial^23^. Patients were able to print out their personalised risk score using the web-based AI-enabled decision aid, *RealRisks®*, and discuss this with their healthcare provider during their consultation; however, this assumes that patients had a suitable device and also had access to a printer. Devices were suggested to be available for patients in clinics in Jimbo et al., to overcome the barrier of access. However, concerns were raised by the participants about patient privacy and confidentiality in completing the information in waiting rooms^16^, and about the possibility of theft and damage to these devices in another study^22^. In a study by Harrison et al., surgeons could only use a tool if they were proficient in English, excluding those who spoke other languages^25^. From these findings, it is clear that AI-enabled decision aids must be designed to prioritise inclusivity, accommodating all patients and clinicians, irrespective of their educational, technological, socioeconomic, or health literacy levels^6,16,17,20,30^.

Predicting the risk or prognosis of a condition generated anxiety for patients in some included studies^8,15,18^. Clinicians’ worried about increasing patients’ anxiety, especially amongst those with a high risk of mortality or morbidity^18^. Patients proposed that a hybrid approach be used, where patients used the decision aid with their clinician during the consultation so as to help reduce their anxiety^15^. This was described by Nelson et al. as a symbiosis between AI and human clinician^15^. The transparency and explainability of AI models is a central component of the Code of Conduct for Data-Driven Health and Care Technology^43^. It is important to understand the real impact of data-driven technologies on patients’ lives, and also have clear expectations regarding the explainability of outputs which are understood by clinicians before, during and after deployment^44^.

Clinicians perceived some AI-enabled decision aids e.g., ACS calculator, u-SHARE as assisting with the patient consent process for surgical procedures; it provided a more accurate personalised risk of post-operative complications following surgery, which was felt to improve patients understanding^12,26^. One hundred and twenty-two patients expressed a desire to use the ACS calculator prior to surgery, with fifty eight considering postponing their surgery until after they had participation in the prehabilitation programme to help reduce their post-operative complication risk^12^. Through reduction of post-operative complications, these tools could potentially reduce the length of hospitalisation and subsequently healthcare costs.

This review highlighted how patients’ and clinicians’ perceptions appeared to vary on the same tool. Although patients often found AI-enabled decision aids user-friendly and valuable^6,20,26,28,30^, clinicians held some reservations about their ease-of-use and whether the information was up-to-date^16,17,20^. These factors were highlighted in a recent literature review as they could act as functional barriers and contribute to patients’ and clinicians’ resistance in using the aids^45^. The information provided by AI-enabled decisions aids needs to be regularly updated once deployed in the clinical setting, and both patients and clinicians need to be informed of any changes, so as to help increase their trust in the tool^46^. Our findings are helpful to a wide variety of individuals and organizations that use and/or develop patient decision aids, and could help inform future updates of IPDAS (International Patient Decision Aid Standards instrument) acknowledging the importance of digital inclusion^47,48^. These updates could also consider the associated risk of bias specifically relating to AI-enabled decision aids, and the potential for over- and under-treatment. The included studies in this review had some limitations. Some clinicians’ perspectives were provided on simulated tools, such as the tool used to aid antibiotic prescribing for GPs^14^, which may have performed differently when deployed in clinical settings^14,33^. Most of the studies (*n* = 22) included in this review were obtained from the use of tools in the medical setting. It was unclear from the included studies whether a formal risk of bias assessment was conducted. For example, some training datasets might have contained data that was unrepresentative or contain socio-historical bias(es) in how the data was entered and collected. It is important to make users aware of any bias(es) and its potential impact on outputs. Future research could explore whether patients’ and clinicians may changes their’ perceptions about the use of AI decision aids when bias mitigation techniques/frameworks, such as PROBAST and QUIPS (Quality in prognostic studies), have not been used or considered in their development. Only studies that included the perspectives of clinicians and/or patients on the use of AI-enabled decision aids in SDM were included; these perspectives are likely to be rare in journals focused on AI advances. The actual development and testing of these decision-aids was outside the scope of this review, so technical databases (i.e. IEEEXplore) were not searched. Evaluating the influence of AI-enabled decision aids on SDM through clinical outcomes in both medical and surgical settings also necessitates real-world testing in clinical practice.

In conclusion, patients found AI-enabled decision aids to be understandable, user-friendly, and empowering. Certain barriers were also acknowledged by both clinicians and patients, in particular concerns related to bias, technological skills, anxiety, and data privacy. These specific barriers must be carefully considered during the development and deployment of future AI-enabled decision aids, so as to ensure that they have a positive impact on patients’ adherence and help inform the shared decision process.

## Methods

This review was registered with The International Prospective Register of Systematic Reviews (PROSPERO) (CRD42020220320) and aligned with the Preferred Reporting Items for Systematic Reviews and Meta-Analyses (PRISMA) guidelines^49^.

### Eligibility criteria

The population, intervention, comparison, outcomes and study (PICOS) design tool recommended by the Cochrane Handbook for Systematic Reviews^50^, (Table 3) was used as an organising framework to list the terms by the main concepts in the search question. We defined an *AI-enabled* decision aid as any data-driven decision aid, developed using machine learning algorithms, that identified patterns and correlations between different variables from individualised patient information and produced tailored recommendation(s) by considering their preferences, medical history and current clinical conditions^51^. All included studies needed to clearly specify that it was *AI-enabled*, with machine learning used to develop the decision aid. All quantitative, qualitative and mixed-method studies needed to also include the perspectives of clinicians (including physicians, pharmacists, and nurses) and/or patients on the use of AI-enabled decision aids to support SDM. A decision aid was judged to inform SDM if it was used between patients and clinicians to facilitate a discussion and/or joint decision^1^. If the tool was used separately by clinicians and/or patients without any discussion and/or joint decision, it was excluded. Only articles published in the English language were included, with no restrictions on the medical speciality, clinical setting, or period over which these decision aids were used.

**Table 3:** Population, Intervention, Comparison, Outcomes and Study (PICOS) framework

Study	Studies that used either quantitative, qualitative or mixed-method studies (i.e. model development and perceptions exploration).
Population	Adults: Patients and Clinicians.
Intervention	Use of an AI-enabled decision aid during the study.
Comparison	N/A (studies did not contain a control group)
Outcomes	Clinicians’ and patients’ perceptions on AI-enabled decision aids to guide future development of these tools.

### Information sources and search strategy

Four large databases were searched, including MEDLINE and Embase (via Ovid), Cumulative Index to Nursing and Allied Health Literature (CINAHL), and SCOPUS. The search was carried out across two timeframes to include published articles from the date of commencement of each database up until Oct 2020, and updated again from Oct 2020 to February 2022. Relevant keywords relating to perceptions, personnel (clinicians/patients), AI and machine learning, algorithms/decision aids, and SDM were used and grouped into sets (see Supplementary Note 1). To ensure coverage, context-specific ‘synonyms’ for the concepts were kept broad. Each set was combined with an “OR” operator, followed by combining all results with an “AND” operator. The search strategy and the keywords are described in Supplementary Note 1.

### Study selection

**Fig. 3:**
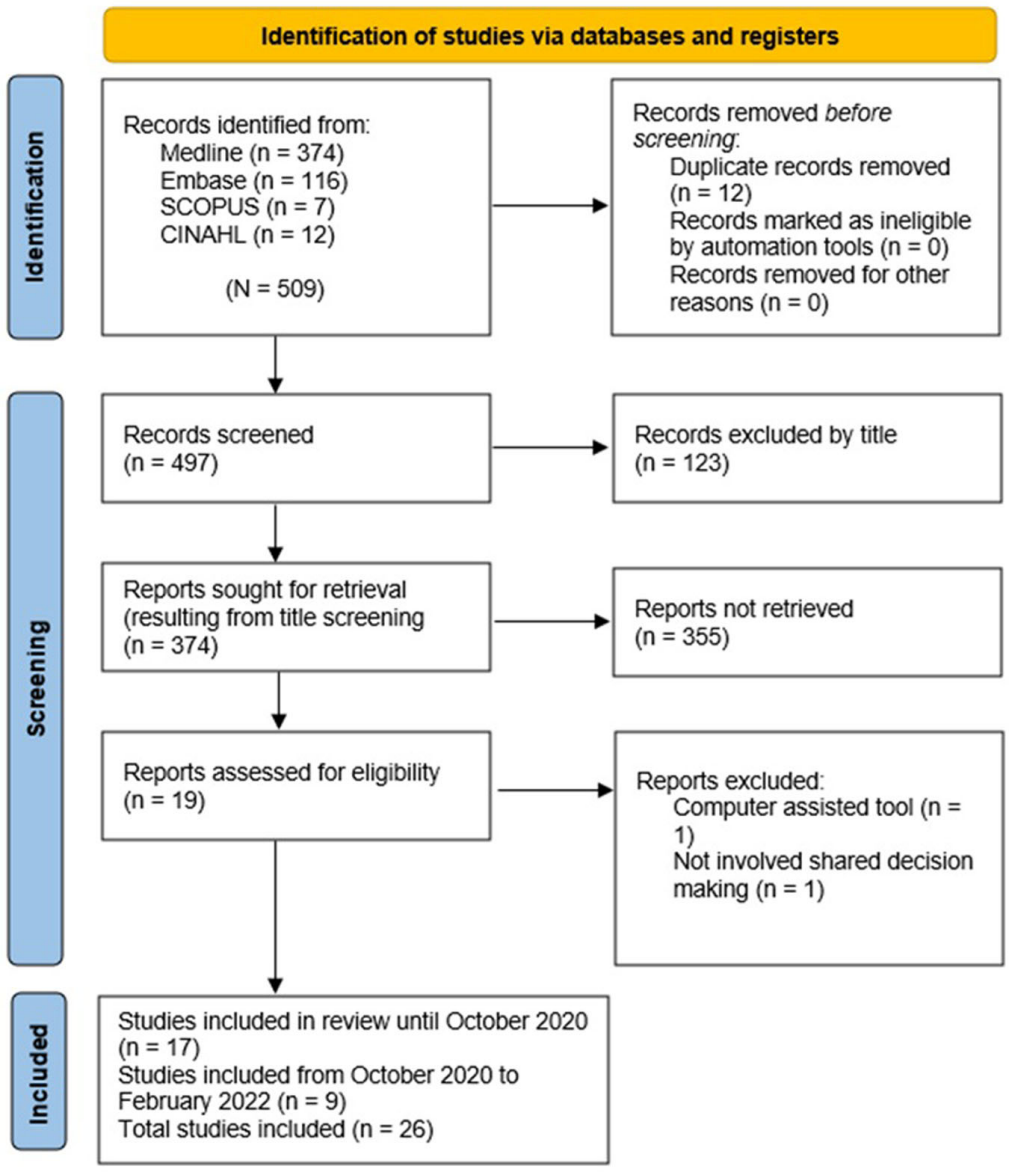
Search strategy PRISMA flowchart. Description of the PRISMA flowchart showing the databases searched and number of articles retrieved at each screening stage.

Duplicates were removed using Endnote software (version X9, Clarivate Analytics, Philadelphia, United States) and manually checked. The corresponding authors of two abstracts were contacted to ask whether there was a complete manuscript available for their studies. Two independent reviewers (NH and KB) screened all titles, abstracts and full texts to determine if they met the inclusion criteria, with any disagreements resolved by discussion with a third reviewer (SPS), if necessary. The reason(s) for excluding any full-text articles were also documented. Inter-rater reliability among the independent reviewers was found to be 100% after discussing disagreements with a third reviewer (SPS). References of the included full-text studies were also screened to identify any potential articles. The search strategy is described in Fig. 3.

### Data extraction and analysis

A customised data-extraction sheet was developed to extract specific details from each study, including study title; year of publication; country; publication type; aim; design; type of user (patients/healthcare professionals); methods; ethical approval; population; clinical setting; medical/surgical speciality; name, type and use of the AI-enabled decision aids and how it contributed to SDM; any AI methodologies utilised, performance metrics, and if potential biases were considered; users’ perceptions; inclusion and exclusion criteria; method of recruitment; and key conclusions. Quantitative data on the number of study participants, percent with a particular perception, and/or the percent who were satisfied/dissatisfied was also extracted.

Data extraction followed the ENhancing Transparency in REporting the synthesis of Qualitative research (ENTREQ) guidelines^52^. The utilised ENTREQ checklist is available in Supplementary Table 1. A narrative synthesis was carried out using the Research Methods Programme framework (ESRC), which accommodates the inclusion of a wide range of research designs^53^. The extracted data was organised and decision aids grouped by their main purpose and where in the patient pathway they were used. Clinicians’ and patients’ perceptions on these different AI-enabled decision aids were then compared.

### Quality assessment

The quality of included articles was assessed using the Critical Appraisal Skills Programme (CASP) tool for qualitative studies^54^. The tool consists of ten questions, with each “Yes” answer given one point. The total score was calculated. All included studies had a score of seven or higher on the CASP checklist (see Supplementary Table 2). No studies were excluded at the stage of quality assessment. As the aim of this review was to understand clinicians’ and patients’ perceptions on using AI-enabled decision aids, specific details around the actual development and validation of such decision aids were not included. It was therefore considered unsuitable to apply the Prediction model Risk Of Bias ASsessment Tool (PROBAST) to assess the risk of bias (ROB) during the development and validation of such model many of which did not contain a prognostic element^55^.

## Supplementary information

Supplementary Information

## Data Availability

All the data generated from this review are included in the manuscript and Supplementary material.
